# Hematopoietic stem cell transplantation-associated thrombotic microangiopathy and the role of advanced practice providers and pharmacists

**DOI:** 10.1038/s41409-023-01951-3

**Published:** 2023-04-14

**Authors:** Zahra Mahmoudjafari, Maritza C. Alencar, Maurice D. Alexander, Darren J. Johnson, Jason Yeh, Misty D. Evans

**Affiliations:** 1 Department of Pharmacy, University of Kansas Cancer Center, Kansas City, KS USA; 2 Oncology Service Line, University of Miami, Sylvester Comprehensive Cancer Center, Miami, FL USA; 3 Department of Pharmacy, University of North Carolina Medical Center, Chapel Hill, NC USA; 4 Pediatric Hematology and Oncology, Dana Farber Cancer Institute, Boston, MA USA; 5 Division of Pharmacy, MD Anderson Cancer Center, Houston, TX USA; 6 School of Nursing, Vanderbilt University, Nashville, TN USA; 7 Sarah Cannon Pediatric Hematology/Oncology & Cellular Therapy at TriStar Centennial, Nashville, TN USA

**Keywords:** Haematological diseases, Diagnosis, Therapeutics

## Abstract

Hematopoietic stem cell transplantation-associated thrombotic microangiopathy (HSCT-TMA) is a severe and potentially life-threatening complication. HSCT-TMA is often underdiagnosed due to multifactorial pathophysiology and a historic lack of standard diagnostic criteria. Identification of the multi-hit hypothesis and the key role of the complement system, particularly the lectin pathway of complement, has led to development of treatments targeting the underlying pathogenesis of HSCT-TMA. Additional research is ongoing to investigate the efficacy and safety of these targeted therapies in patients with HSCT-TMA. Advanced practice providers (APPs; nurse practitioners and physician assistants) and pharmacists are critical members of the multidisciplinary HSCT team and ensure management of patients throughout the continuum of care. Additionally, pharmacists and APPs can improve patient care through medication management of complex regimens; transplant education for patients, staff, and trainees; evidence-based protocol and clinical guideline development; assessment and reporting of transplant-related outcomes; and quality improvement initiatives to improve outcomes. Understanding the presentation, prognosis, pathophysiology, and treatment options for HSCT-TMA can improve each of these efforts.

**Figure:**
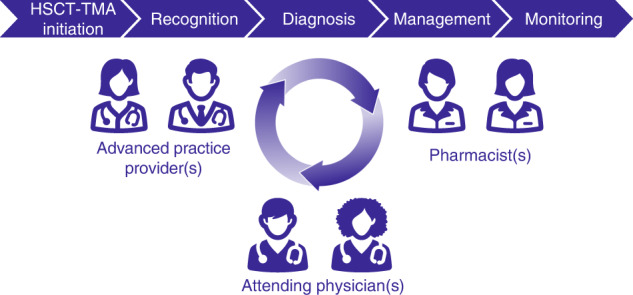
Collaborative practice model for monitoring and care of HSCT-TMA. Advanced practice providers and pharmacists contribute to many aspects of patient care in transplant centers, including medication management for complex regimens; transplant education for patients, staff, and trainees; evidence-based protocol and clinical guideline development; assessment and reporting of transplant-related outcomes; and quality improvement initiatives. HSCT-TMA is a severe and potentially life-threatening complication that is often underdiagnosed. The collaboration of a multidisciplinary team of advanced practice providers, pharmacists, and physicians can optimize recognition, diagnosis, management, and monitoring of patients with HSCT-TMA, thereby improving outcomes for these patients.

Collaborative practice model for monitoring and care of HSCT-TMA. Advanced practice providers and pharmacists contribute to many aspects of patient care in transplant centers, including medication management for complex regimens; transplant education for patients, staff, and trainees; evidence-based protocol and clinical guideline development; assessment and reporting of transplant-related outcomes; and quality improvement initiatives. HSCT-TMA is a severe and potentially life-threatening complication that is often underdiagnosed. The collaboration of a multidisciplinary team of advanced practice providers, pharmacists, and physicians can optimize recognition, diagnosis, management, and monitoring of patients with HSCT-TMA, thereby improving outcomes for these patients.

## Role of advanced practice providers and PharmaciSts in HSCT and HSCT-TMA

Hematopoietic stem cell transplantation (HSCT) is the only curative treatment option for many patients with life-threatening hematologic and oncologic illnesses. Nearly 24,000 stem cell transplants are performed each year in the United States alone [1]. Appropriate management of this complex patient population requires providers who are knowledgeable and well-trained in this specialty area. Pharmacists and advanced practice providers (APPs; nurse practitioners and physician assistants) are critical members of the multidisciplinary HSCT team who play an essential role in the management of patients throughout the continuum of care. They are often responsible for the early recognition, diagnosis, or management of post-HSCT complications. Additionally, pharmacists and APPs frequently contribute to hematology-oncology and HSCT centers to improve patient care such as the following: medication management for complex regimens; transplant education for patients, staff, and trainees; evidence-based protocol and clinical guideline development; assessment and reporting of transplant-related outcomes; and quality improvement initiatives to improve outcomes. The purpose of this review is to summarize the role of pharmacists and APPs in the identification and management of HSCT-TMA, specifically the clinical presentation, diagnosis, consequences, prognosis, pathophysiology, and available therapies for HSCT-TMA.

Pharmacists and APPs involved in the care of patients after HSCT must be well educated and trained in HSCT to provide quality care and positively impact outcomes. At large medical centers, the multidisciplinary care team comprising physicians, APPs, and pharmacists review available literature and make recommendations on diagnostic parameters and treatment options to create a standardized algorithm (pathway) to guide patient care and manage toxicities. Once this algorithm is created, it is then routinely updated based on new literature or clinical experience. Ensuring a standard protocol is followed ensures consistency among team members. These protocols typically have capacity for patient-specific factors and are an optimal approach to diagnose and manage significant posttransplant complications. Pharmacists and APPs may participate in formulary management decisions for therapies used to treat HSCT-TMA. Availability of therapies for HSCT-TMA can be particularly challenging due to limited local supply and required Risk Evaluation and Mitigation Strategy programs for prescribers. Having knowledgeable and resourceful APPs and pharmacists can help provide reasonable expectations and expedite procurement in these high-acuity situations.

Organizations such as the American Society of Transplantation and Cellular Therapy (ASTCT), the European Society of Blood and Marrow Transplantation (EBMT), and the National Marrow Donor Program (NMDP) support specialized training and competency for APPs and pharmacists [2–5]. Additionally, Edition 8 of the FACT-JACIE (Foundation for the Accreditation of Cellular Therapy–Joint Accreditation Committee of the International Society for Cell and Gene Therapy and the European Society for Blood and Marrow Transplantation) International Standards for Hematopoietic Cellular Therapy Product Collection, Processing, and Administration issued in May 2021 delineates the roles and responsibilities for each member of the team [6]. The responsibilities for all care providers, including APPs and pharmacists, require specific training in complications of HSCT. These standards list numerous events requiring specific training and monitoring, such as neutropenic fever, pulmonary complications, sinusoidal obstruction syndrome, thrombocytopenia and bleeding, gastrointestinal complications, and neurological complications (among others).

**Fig. 1:**
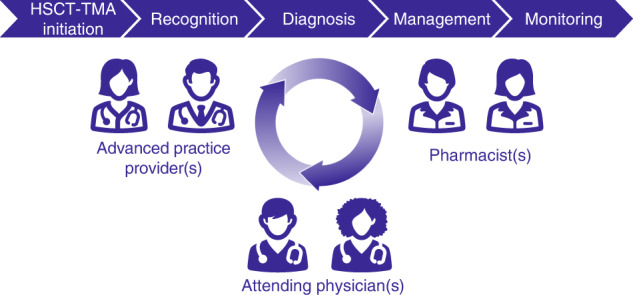
Collaborative practice model for monitoring and care of HSCT-TMA. Advanced practice providers and pharmacists contribute to many aspects of patient care in transplant centers, including medication management for complex regimens; transplant education for patients, staff, and trainees; evidence-based protocol and clinical guideline development; assessment and reporting of transplant-related outcomes; and quality improvement initiatives. HSCT-TMA is a severe and potentially life-threatening complication that is often underdiagnosed. The collaboration of a multidisciplinary team of advanced practice providers, pharmacists, and physicians can optimize recognition, diagnosis, management, and monitoring of patients with HSCT-TMA, thereby improving outcomes for these patients.

HSCT-associated thrombotic microangiopathy (HSCT-TMA) is a severe and potentially life-threatening complication that is often underdiagnosed due to the complex, multifactorial pathophysiology and lack of a standard set of diagnostic criteria [7–9]. To address this gap, the ASTCT, EBMT, Center for International Bone Marrow Transplant Research (CIBMTR), and Asia-Pacific Blood and Marrow Transplantation (APBMT) societies recently published harmonized definitions for HSCT-TMA diagnostic and prognostic criteria [10]. The adoption and implementation of these emerging definitions in clinical practice remain to be determined. Notably, HSCT-TMA is not listed as one of the complications requiring specific training in the 2021 edition of the FACT-JACIE standards [6]. It is therefore the responsibility of APPs and pharmacists to seek additional information on this topic, particularly as ongoing studies of targeted therapies are completed, and new therapies are approved for this indication. To improve the prognosis and outcomes for patients with HSCT-TMA, APPs and pharmacists must take a leading role in the early recognition, diagnosis, and development of evidence-based treatment plans for these patients (Fig. 1).

## Clinical presentation and diagnosis

HSCT-TMA has a reported incidence of 4–68% in adults [7–9, 11–15] and 3–39% in children after HSCT [16–19]. HSCT-TMA is often underdiagnosed, with one study reporting confirmed diagnoses in fewer than half of patients with suspected cases [9]. This is particularly evident in real-world clinical practice, where fewer than 1% of pediatric patients receiving HSCT are diagnosed with HSCT-TMA [20].

A historic lack of consensus on diagnostic criteria has been a likely contributor to the wide range of incidences reported in clinical research [20, 21]. Several clinical signs have been proposed as supporting a diagnosis of HSCT-TMA, including thrombocytopenia, hemolytic anemia, absence of antibodies, tissue damage, kidney dysfunction, and hypertension (Table 1) [19, 22–26]. Many of these are non-specific clinical signs and symptoms that occur frequently post-transplant and that could be medication-related or due to alternative post-transplant syndromes. Some signs such as proteinuria, hypertension, and elevated lactate dehydrogenase (LDH) are quite common in HSCT-TMA and can occur earlier (a median of 10 to 14 days before the diagnosis of HSCT-TMA [19]), while others such as schistocytes and elevated serum creatinine tend to occur later and less often.

**Table 1:** Diagnostic criteria for HSCT-TMA.

Source, year	Tissue damage	Hemolytic anemia			No antibodies	Thrombocytopenia	Kidney dysfunction		Hypertension	Other
	LDH ↑	Schistocytes	Hemoglobin ↓	Haptoglobin ↓	Coombs test (–)	Platelets ↓	Serum creatinine ↑	Proteinuria	Blood pressure ↑	
BMT CTN (2005) [22]	√	√			√		√			(1)
IWG (2007) [23]	√	√	√^a^	√		√				
Cho et al. (2010) [24]	√	√	√	√	√	√				
City of Hope (2013) [25]	√	√				√	√			
ASH-EBMT (2014) [26]	√	√	√		√	√		√	√	(2, 3)
Jodele et al. (2014) [19]	√	√	√^a^			√		√	√	(2, 4)
Schoettler et al. (2022) [10]	√	√	√^b^			√^c^		√	√	(2, 4)

*ASH-EBMT* American Society of Hematology–European Society for Blood and Marrow Transplantation, *BMT CTN* Blood and Marrow Transplant Clinical Trials Network, *IWG* International Working Group, *LDH* lactate dehydrogenase, *TMA* thrombotic microangiopathy.

Other: (1) neurologic dysfunction; (2) elevated soluble C5b-9; (3) transplant-associated index (ratio between LDH and platelets) ≥20; (4) must meet at least 4 criteria at 2 consecutive time points within 14 days.

^a^Decreased hemoglobin or increased red blood cell transfusion requirement.

^b^Failure to achieve transfusion independence for packed red blood cells despite evidence of neutrophil engraftment; hemoglobin decline from baseline by 1 g/dL; or new onset of transfusion dependence. Rule out other causes as sole cause of anemia.

^c^Failure to achieve platelet engraftment; higher than expected platelet transfusion needs; refractoriness to platelet transfusions; or a 50% reduction in baseline platelet count after full platelet engraftment.

Routine testing has been proposed to screen for HSCT-TMA, including a daily complete blood count, twice-weekly LDH, weekly urinalysis, and routine blood pressure [19]. Even when patients are monitored closely, cases that would be identified by laboratory criteria may not be diagnosed as HSCT-TMA [8]. Signs and symptoms of HSCT-TMA can be mistaken for other common complications such as graft-versus-host disease (GVHD), infection, or medication-induced hypertension or kidney dysfunction [27]. HSCT-TMA often becomes a diagnosis of exclusion [28]. Laboratory testing to rule out other diagnoses includes the ADAMTS13 enzyme (for thrombotic thrombocytopenic purpura), coagulation studies (for disseminated intravascular coagulation), direct Coombs test (for autoimmune hemolytic anemia), T-cell chimerism (for GVHD), or viral polymerase chain reaction (for infection) [21].

The variable time to onset of HSCT-TMA is another diagnostic challenge. HSCT-TMA can occur within days of HSCT, particularly in pediatric patients, but it often occurs weeks or months after HSCT, when patients are monitored less rigorously for post-transplant complications. Two large studies reported a median time to onset of 57 and 86 days (approximately 2–3 months), with time to onset for some patients that exceeded 1 year in each study [29, 30]. A smaller study of early and late HSCT-TMA reported that approximately half of the cases occurred late (more than 100 days after HSCT), with a median of 303 days and a maximum of 2595 days after transplant for these late HSCT-TMA cases [31]. The potential for late presentation of HSCT-TMA underscores the importance of long-term monitoring for this complication. HSCT-TMA should be considered in the differential diagnosis for any nonspecific complication that can initially appear to be infection or another complication.

Tissue biopsy is the most reliable method to confirm HSCT-TMA. Histologic analysis of kidney biopsy in a patient with HSCT-TMA shows microthrombi in glomeruli and C4d deposition in renal arterioles [32]. Biopsy can also be used to confirm the diagnosis of HSCT-TMA in the gastrointestinal tract [33]. However, kidney biopsy and gastrointestinal biopsy are high-risk procedures, particularly after HSCT in the setting of thrombocytopenia. Thus, in addition to the broad panel of blood tests that has been recommended for diagnostic criteria (Table 1), biomarkers have been investigated for their ability to identify HSCT-TMA or predict outcomes in patients with HSCT-TMA (Table 2). Markers for neutrophil activation have been shown to distinguish cases of HSCT-TMA from cases of GVHD [34]. Elevations of suppressor of tumorigenicity 2 (ST2) after HSCT are associated with an increased risk of HSCT-TMA when ST2 is measured either alone or in combination with regenerating islet-derived 3α (REG3α) [35, 36]. Some reports have shown that complement system proteins such as C5b-9 (discussed in greater detail below) are increased in the plasma of patients with HSCT-TMA [19, 34]. However, other reports have suggested that plasma levels of complement proteins do not increase, even when endothelial cells show evidence of C5b-9 deposition [37]. Elevated serum C5b-9 has been identified as both a diagnostic biomarker and a high-risk prognostic feature in HSCT-TMA; however, the test is not widely available [10]. Mannan-binding lectin-associated serine protease-2 (MASP-2) levels are highly elevated in patients with HSCT-TMA compared with controls [38]. Antibodies to Factor H in the alternative pathway have been reported to occur selectively in patients who develop HSCT-TMA [39]. The Endothelial Activation and Stress Index (EASIX)—combining LDH, creatinine, and platelets—is higher in patients with HSCT-TMA or GVHD compared with controls [40]. EASIX scores have a strong, positive association with C5b-9 serum concentrations, and higher EASIX scores are negatively associated with survival [40]. These biomarkers have not yet been incorporated into routine clinical care and work up of HSCT-TMA as they are specialized assessments that are often not routinely available at institutional laboratories. This can necessitate the use of referral laboratories, delaying results and subsequent treatment. Additional studies are needed to clarify the utility of these biomarkers for the diagnosis, management, and prognosis of HSCT-TMA, to support their routine use in clinical practice.

**Table 2:** Potential biomarkers for occurrence or prognosis of HSCT-TMA.

Potential biomarker	Diagnostic indicator	Indicator of poorer prognosis
Neutrophil activation [34]	+	
ST2 [35]	+	
ST2 + REG3α [36]	+	
C5b-9 [19, 40]	++	+
EASIX [40]	++	+
MASP-2 [38]	++	
Factor H antibodies [39]	+	

*+* positive association reported, *++* strong positive association reported, *EASIX* Endothelial Activation and Stress Index, *MASP-2* mannan-binding lectin-associated serine protease-2, *REG3α* regenerating islet-derived 3α, *ST2* suppressor of tumorigenicity 2.

## HSCT-TMA clinical consequences and prognosis

**Fig. 2:**
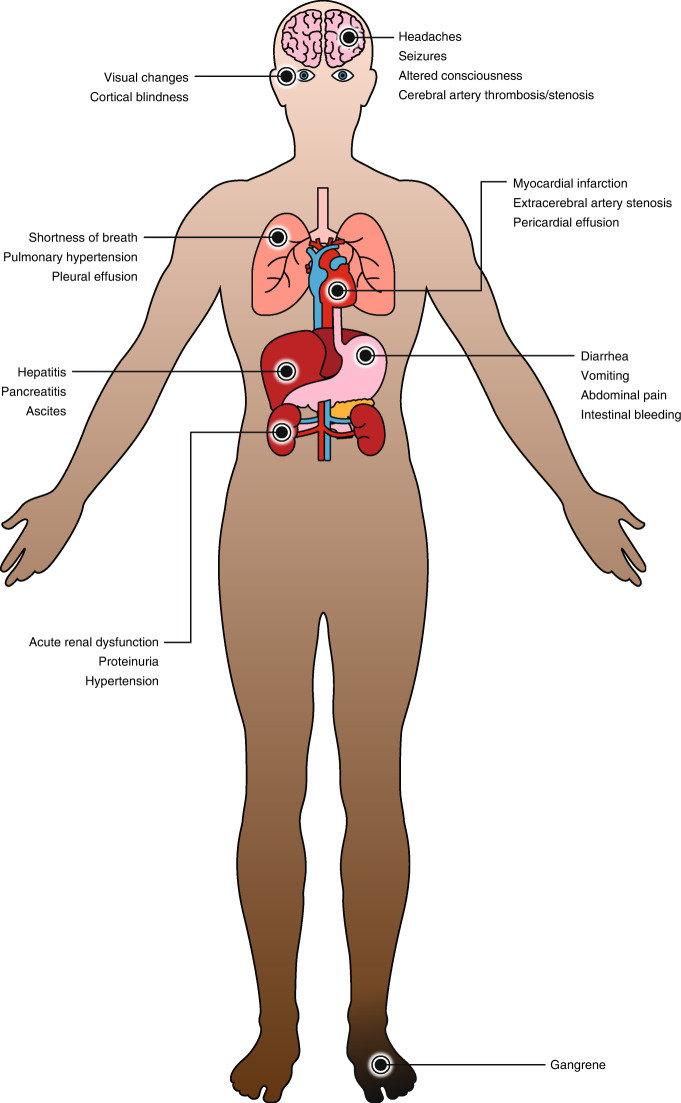
Possible organ involvement in HSCT-TMA. HSCT-TMA often presents as acute kidney dysfunction, but other organs are involved in up to 20% of patients [21, 41].

In patients with HSCT-TMA, endothelial injury resulting from HSCT leads to microthrombus formation, microangiopathic hemolytic anemia, thrombocytopenia, and organ damage due to deposition of microthrombi in the small vasculature of affected organs [21]. HSCT-TMA often presents as acute kidney dysfunction, but other organs are involved in up to 20% of patients, including neurologic involvement (seizures, altered consciousness), pancreatitis, cardiac involvement (myocardial infarction), gastrointestinal involvement (diarrhea, vomiting, abdominal pain), cerebral artery thrombosis/stenosis, extracerebral artery stenosis, digital gangrene, ocular involvement, hepatitis, and pulmonary involvement (Fig. 2) [21, 41].

Non-relapse mortality has been reported to occur in up to 75% of patients with HSCT-TMA [27, 42] and is higher (>90%) among patients with severe HSCT-TMA and multiorgan involvement [27]. A diagnosis of HSCT-TMA increases transplant-related mortality more than threefold overall [43] and fivefold in children [44]. Improvement of HSCT-TMA has been shown to significantly improve survival [31], but a patient who survives HSCT-TMA remains at increased risk of permanent organ damage such as chronic kidney disease [45].

Among patients with HSCT-TMA, several risk factors for poor prognosis have been identified, including age ≥18 years, donor type (unrelated or haploidentical), an elevated LDH or LDH/platelet ratio, proteinuria, kidney dysfunction requiring dialysis, any other organ dysfunction that develops during HSCT-TMA (except mild acute kidney dysfunction), concurrent GVHD, and concurrent systemic infection [10, 19, 27, 46, 47]. As discussed above and summarized in Table 2, serum C5b-9 and EASIX may also be markers for worse prognosis in HSCT-TMA [10, 19, 40].

## Pathophysiology

**Fig. 3:**
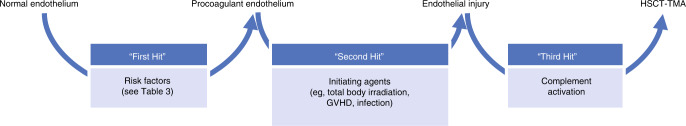
Multi-hit hypothesis of HSCT-TMA pathogenesis [47, 48]. The three-hit hypothesis [48] and two-hit hypothesis [47] both postulate that risk factors (first hit) and initiating agents (second hit) predispose a patient to HSCT-TMA. The third hit (complement activation), which is sometimes considered an extension or consequence of the second hit, appears to be an essential step in the pathogenesis of HSCT-TMA.

The pathogenesis of HSCT-TMA has been described using either a two-hit or three-hit hypothesis, as reviewed in detail previously [47, 48]. A brief overview is provided here as a framework for the prevention, early diagnosis, and treatment of HSCT-TMA. Both models share the “first hit” of underlying patient predisposition, and the “second hit” of endothelial injury. The three-hit model further postulates that continued endothelial injury due to medications, alloreactivity, infection, or antibody formation triggers activation of the complement system (Fig. 3).

For the first hit, several risk factors for development of HSCT-TMA and for poorer prognosis have been identified (Table 3), but not all risk factors are modifiable [46, 47]. For example, nonmodifiable genetic variants in complement system proteins that increase complement activity and increase the risk of HSCT-TMA have been reported in children [49, 50] and in adults [51]. The second hit of endothelial injury results from various causative factors that are common among patients undergoing HSCT and that overlap with known risk factors. Endothelial injury can be initiated by total body irradiation, GVHD, or infection [47, 52]. Most of these causative factors of endothelial injury are associated with early HSCT-TMA (within 100 days) but chronic GVHD is significantly more likely to be associated with late HSCT-TMA (more than 100 days after HSCT) [31].

**Table 3:** Risk factors for procoagulant endothelium and development of HSCT-TMA [10, 19, 27, 46, 47, 49, 51].

Risk factors for development of HSCT-TMA
Donor type
Prolonged immobilization
Severe bacterial and fungal infections
Endothelial injury (by conditioning or GVHD)
Indwelling central venous catheters
Residual leukemic cells present at the time of transplant
Growth factor administration
Venous thromboembolism
Unrelated donor transplants
Non-myeloablative transplants (fludarabine-based regimens)
High-dose busulfan (16 mg/kg)
HLA mismatch (one or more loci)
Female sex
Genetic variants

*GVHD* graft-versus-host disease, *HLA* human leukocyte antigen, *LDH* lactate dehydrogenase.

There is extensive overlap between GVHD and HSCT-TMA, including triggers, the presence of endothelial damage, and clinical presentation, making it very difficult to distinguish between the two conditions [29]. A major distinction in pathogenesis appears to be activation of the complement system in patients with HSCT-TMA, based on a significant increase in the end products of complement activation that is not seen in patients with GVHD alone [34]. Thus, complement system activation has been proposed either as the mediator of the “second hit” in HSCT-TMA (endothelial injury) [47], or as a distinct “third hit” resulting from the first and second hits [48].

## Activation of complement in HSCT-TMA

Complement, a vital part of the body’s innate immune response, consists of more than 30 proteins and glycoproteins that are formed predominantly in the liver and enter the circulation in their inactive forms [53]. Inactive components are converted to active forms to enhance the immune response to infection or injury. Key activation products of the complement system include C3a and C5a, C3b, and C5b-9. C3a and C5a are anaphylatoxins that predominantly stimulate inflammation, but can also stimulate thrombosis, leukocyte recruitment, and endothelial cell activation. C3b binds to glycoproteins on target cells, opsonizing and presenting the target cells to phagocytic cells for destruction. C5b-9, also known as the membrane attack complex, forms trans-membrane channels on target cells, leading to apoptosis.

**Fig. 4:**
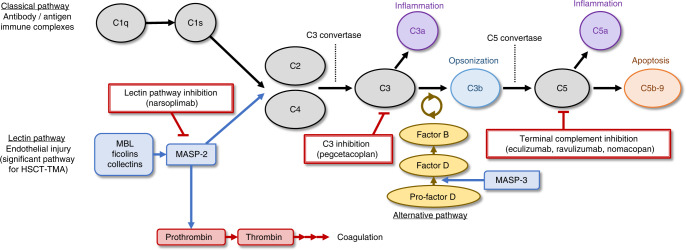
Role of the complement system in HSCT-TMA. In the classical pathway, C1q binds to the antibody complex, activating C1s. In the lectin pathway, carbohydrate patterns on injured endothelial cells are recognized by pattern-recognition molecules that activate MASP-1 and MASP-2. C1s (from the classical pathway) or MASP-2 (from the lectin pathway) cleave C2 and C4 to C2b and C4b, among other products. MASP-1 also cleaves C2. The C4b2b complex, or “C3 convertase,” cleaves C3 to form C3a and C3b [54]. The C3bC4b2b complex, or “C5 convertase,” cleaves C5 to C5a and C5b, initiating the terminal pathway to form the C5b-9 membrane attack complex [53]. The alternative pathway amplifies the classical and lectin pathways. C3 is labile and spontaneously hydrolyzes to C3(H_2_O) at a low but constant rate, through “C3 tickover” [57]. MASP-3 converts pro-Factor D to Factor D, which cleaves Factor B into Ba and Bb. Hydrolyzed C3 and Bb combine to cleave C3, which produces C3b and contributes to an amplification loop via formation of the alternative pathway “C3 convertase,” or C3bBb. MBL mannan-binding lectin; MASP-2 mannan-binding lectin-associated serine protease-2.

In general, the complement system is activated by three major pathways: the classical, lectin, and alternative pathways (Fig. 4). Initiation of the classical pathway occurs in response to antibodies bound to surface antigens, leading to multiple forms of immunity: inflammation (C3a, C4a, C5a), opsonization (C3b), and direct lysis of cells (C5b-9) [53, 54]. Endothelial injury associated with HSCT initiates the lectin pathway, which is thought to be a significant pathway for activation of the complement system in patients with HSCT-TMA. In endothelial injury, the lectin pathway is activated in response to carbohydrate patterns displayed on injured cells that are recognized by pattern-recognition molecules such as mannan-binding lectin, collectins, and ficolins. These bound pattern-recognition molecules form activation complexes with MASPs [53]. Activated MASP-1 and MASP-2 cleave C2 and C4 (both cleave C2; MASP-2 also cleaves C4 [55, 56]), leading to the downstream effects on the complement system. The alternative pathway activates complement through an amplification loop in which spontaneously activated C3 leads to further C3 cleavage [53, 57]. The alternative pathway also amplifies responses to the classical or lectin pathways, which have already generated C3b.

The importance of the complement system in the third hit is supported by research showing that C3b and C5b-9 levels are elevated in patients with HSCT-TMA, and increases in these complement components are associated with increased mortality rates [19, 58]. Because HSCT-TMA results from endothelial injury, the lectin pathway is primarily responsible for activation of the complement system in patients with HSCT-TMA. Further evidence supporting the key role of lectin pathway activation includes research showing patients have increased MASP-2 levels after HSCT [38]. In addition to its effects on the complement system, MASP-2 has been shown to convert prothrombin to thrombin, directly contributing to the coagulation cascade [59].

## Supportive therapy

Several treatments have been recommended for supportive care (Table 4) [21], but none are approved for the treatment of HSCT-TMA and no national or international treatment guidelines are available. Therapeutic plasma exchange is often used to manage thrombotic thrombocytopenic purpura [60]. Although therapeutic plasma exchange is generally well tolerated, there is weak evidence to support its use in patients with HSCT-TMA [60, 61]. Indeed, recent evidence shows that therapeutic plasma exchange does not decrease the risk of chronic organ damage in these patients [62]. Thus, therapeutic plasma exchange is suggested for patients with complement-mediated TMA when complement-targeting therapy is not available [60]. Another supportive treatment for HSCT-TMA, defibrotide is approved for the treatment of hepatic veno-occlusive disease, also known as sinusoidal obstruction syndrome, a complication following HSCT [63]. Retrospective analyses and case series of defibrotide therapy have reported responses in patients with HSCT-TMA [46, 64–67] and a prospective pilot study suggested prophylactic defibrotide use reduces the incidence of HSCT-TMA [68], but increased bleeding and hemorrhagic complications were reported with defibrotide use in these studies [65–68]. Historically, another approach to treat HSCT-TMA has been to withhold or modify calcineurin inhibitors [22], but recent data have shown that continuing immunosuppressants does not impact resolution of HSCT-TMA or survival in patients with HSCT-TMA. Resolution rates for HSCT-TMA are similar among patients who have immunosuppressants withdrawn versus those who continue immunosuppressants [8] and response rates and survival are significantly higher among patients who continue a calcineurin inhibitor instead of switching to a corticosteroid [69]. In patients with definite HSCT-TMA, survival rates are also higher among patients with sirolimus exposure versus patients without sirolimus exposure [9].

**Table 4:** Supportive care for HSCT-TMA [21, 48, 68].

Supportive care	Potential benefits	Potential limitations
Therapeutic plasma exchange	Might decrease activated complement components in plasma	Has not been shown to decrease the risk of chronic organ damage in HSCT-TMA
Defibrotide	Responses have been reported	Bleeding complications have also been reported
Manage hypertension	Present in almost all cases of HSCT-TMA	Has not been shown to affect risk or progression of HSCT-TMA
Avoid nephrotoxins	Prevents further damage in patients with kidney involvement	Has not been shown to affect risk or progression of HSCT-TMA
Treat underlying infection	Decreased endothelial injury	Infection is not always present
Manage underlying GVHD	Decreased endothelial injury	Some treatments for GVHD increase the risk of HSCT-TMA

*GVHD* graft-vs-host disease.

Management of common signs and symptoms of HSCT-TMA is also suggested, such as one or more medications to aggressively treat hypertension (when present) or avoidance of common nephrotoxins (when kidney damage is present) such as amphotericin B, foscarnet, sulfamethoxazole-trimethoprim, vancomycin, diuretics, angiotensin-converting enzyme inhibitors, and angiotensin receptor blockers [70]. Bacterial infections, which are commonly seen in patients with severe TMA [71], should be included in the routine workup for HSCT-TMA and treated promptly to reduce the risk of additional endothelial injury. Concurrent GVHD should be treated with corticosteroids or other immunosuppressive agents [48].

Optimal supportive care for patients with HSCT-TMA requires both an understanding of evolving evidence for appropriate management of HSCT-TMA and close monitoring of how patients respond to that care. Pharmacists and APPs are vital in this setting, accounting for various drug interactions during changes in therapy and monitoring the patient for therapeutic responses and adverse events for new therapies.

## Targeted therapy

Several therapies that directly target the complement system have recently been investigated in clinical trials of patients with HSCT-TMA (Table 5, Fig. 4). To date, no targeted therapy has been approved for use in patients with HSCT-TMA, and many of these trials are still ongoing. Other complement inhibitors currently under development for a variety of complement-mediated disorders may also be investigated for use in patients with HSCT-TMA eventually.

**Table 5:** Complement inhibitors in clinical trials for HSCT-TMA.

Therapy	Mechanism of action	Study identifier	Phase	Population	Control	Status	No. of Patients
Eculizumab	Monoclonal antibody to C5; terminal complement inhibitor	NCT03518203	2	Pediatric/adult	None	Ongoing	*N* ≈ 23
Ravulizumab	Monoclonal antibody to C5; terminal complement inhibitor	NCT04557735	3	Pediatric	None	Ongoing	*N* ≈ 40
		NCT04543591	3	Adolescent/adult	Placebo	Ongoing	*N* ≈ 184
Nomacopan	C5 + leukotriene B4 inhibitor; terminal complement inhibitor	NCT04784455	3	Pediatric	None	Ongoing	*N* ≈ 50
Pegcetacoplan	C3 inhibitor	NCT05148299	2	Adult	None	Ongoing	*N* ≈ 12
Narsoplimab	Monoclonal antibody to MASP-2; lectin pathway inhibitor	NCT02222545	2	Adult	None	Completed [85]	*N* = 28
		EudraCT 2021-002727-38	2	Pediatric	None	Initiation	*N* ≈ 18

*MASP-2* mannan-binding lectin-associated serine protease-2.

### C5 inhibitors (eculizumab, ravulizumab, nomacopan)

The terminal complement inhibitor, eculizumab, is a monoclonal antibody to C5 that blocks the production of the anaphylatoxin C5a and the C5b-9 membrane attack complex, thereby inhibiting two of the major end-products of the complement system [72]. Eculizumab is approved for use in patients with atypical hemolytic uremic syndrome (aHUS), which shares many features with HSCT-TMA [73]. A case series reported that 4 of 6 children with HSCT-TMA who received eculizumab weekly achieved a complete response [74]. A systematic review of case series in 2014 reported that 7 of 9 patients (including the 4 of 6 already described) responded to eculizumab for HSCT-TMA [75]. Responses to eculizumab were also reported in two subsequent case reports for individual patients with HSCT-TMA [76, 77], and in two retrospective case series of 12 or 15 patients, respectively [78, 79]. The latter study reported high response rates but poor long-term survival (33% at 30 weeks), due largely to infection-related mortality (70% of deaths) [79]. The largest retrospective analysis of eculizumab treatment to date reported that among 64 pediatric patients with HSCT-TMA, 64% had measurable responses and the 1-year survival rate was 66% [18]. A prospective Phase 2 study of eculizumab for TMA (or aHUS or multiple organ dysfunction syndrome) after HSCT is ongoing (NCT03518203). For the treatment of aHUS, eculizumab is administered intravenously at weekly doses for the first 5 weeks (or fewer weeks in children with body weight below 40 kg), then every 2 weeks [72]. Supplemental dosing is required in the setting of concomitant plasmapheresis or plasma exchange, or fresh frozen plasma infusion. Eculizumab has a boxed warning for serious meningococcal infections, requiring immunization 2 weeks before the first dose, and eculizumab is available only through a restricted program under a Risk Evaluation and Mitigation Strategy (REMS). If a patient is started on eculizumab and is not a candidate for meningococcal vaccines, antibiotic prophylaxis against encapsulated organisms should be considered for at least 8 weeks after the last dose of eculizumab [80].

Ravulizumab is also a monoclonal antibody to C5, but with a longer terminal half-life than eculizumab [81]. Ravulizumab is approved for the treatment of aHUS in children and adults who are either treatment-naïve or have received eculizumab for at least 3 months and have demonstrated a response to eculizumab. Results of clinical studies of ravulizumab in patients with HSCT-TMA are not yet available, but Phase 3 studies are underway in pediatric patients (NCT04557735) or adolescents and adults (NCT04543591) with HSCT-TMA. After a loading dose, ravulizumab is administered intravenously every 4 or 8 weeks. It is also available only through a REMS and has the same boxed warning as eculizumab for serious meningococcal infections.

Nomacopan (formerly coversin) is a small protein inhibitor of both C5 and leukotriene B4 [82] that is also being investigated in a Phase 3 study of pediatric patients with HSCT-TMA (NCT04784455). Results of clinical studies of nomacopan in patients with HSCT-TMA have not been published. A case study reported that a pediatric patient with HSCT-TMA resistant to eculizumab treatment subsequently responded to daily nomacopan treatment, then progressed and died when nomacopan was switched to alternate-day administration due to limited supply [83].

### C3 inhibitors (Pegcetacoplan)

Pegcetacoplan, which targets the proximal complement protein C3, is approved for the treatment of paroxysmal nocturnal hemoglobinuria [84]. A Phase 2 study of pegcetacoplan in 12 adults with HSCT-TMA was recently initiated (NCT05148299). Results from this ongoing study have not been published.

### Lectin pathway inhibitor (Narsoplimab)

Narsoplimab is a fully human monoclonal antibody targeting MASP-2, the effector enzyme of the lectin pathway [38]. Inhibiting the lectin pathway has potential to decrease activation of complement activation-products contributing to inflammation and end organ damage [19, 55, 56, 58]. Targeting MASP-2 is also expected to inhibit thrombus formation resulting from the coagulation cascade [59]. The efficacy and safety of narsoplimab were investigated in a single-arm, open-label pivotal Phase 2 study of adults with HSCT-TMA who received intravenous narsoplimab once weekly for 4–8 weeks (NCT02222545). Among 28 patients receiving at least one dose of narsoplimab, 17 (61%) had improvement in laboratory TMA markers (platelet count and lactate dehydrogenase) and showed clinical benefit (improvement in organ function or freedom from transfusion). The 100-day survival rate after HSCT-TMA diagnosis was 68%, median overall survival was 274 days, and adverse events were typical of this population, with no apparent safety signal of concern [85]. A Phase 2 study is evaluating the efficacy and safety of narsoplimab in pediatric patients with high-risk HSCT-TMA (EudraCT 2021-002727-38).

### Use of biomarkers to monitor responses to targeted treatment

For treatments targeting the complement system, biomarkers could be useful to assess treatment responses and guide dosing decisions. As discussed above, several biomarkers such as EASIX have been shown to predict occurrence and outcomes for HSCT-TMA [34–36, 38–40], but they do not provide a direct measure of responses to complement-targeted therapy. In a patient with HSCT-TMA, increased levels of activated components such as C5b-9 or activated Factor B (Bb) suggest activation of the complement system, whereas levels tend to be normal or low for inactive components such as Factor B, total hemolytic complement assay (CH50), or complement alternative pathway activity (AH50) [19, 86]. However, these tests are neither specific for HSCT-TMA (for example, low AH50 can also occur in patients with infection) nor sensitive for HSCT-TMA [86]. The use of these and other biomarkers to monitor responses to targeted therapy remains to be confirmed.

## Conclusions

HSCT-TMA remains a significant and underdiagnosed event after transplantation. All members of the multi-disciplinary HSCT team must have a clear understanding of the risk factors, pathophysiology, and potential treatment options for HSCT-TMA to optimally manage this common and potentially severe complication.
